# Empyema Necessitans Due to Tuberculosis in a Patient With Poorly Controlled Diabetes Mellitus Type 2: A Case Report

**DOI:** 10.7759/cureus.73919

**Published:** 2024-11-18

**Authors:** Shehan Silva, Lahiru Waduge

**Affiliations:** 1 Department of Medicine, Faculty of Medical Sciences, University of Sri Jayewardenepura, Colombo, LKA; 2 University Medical Unit, Colombo South Teaching Hospital, Kalubowila, LKA; 3 Medicine, Colombo South Teaching Hospital, Kalubowila, LKA

**Keywords:** empyema necessitans, extra pulmonary tuberculosis, mycobacterium tuberculosis, pleural effusion, tuberculosis

## Abstract

Empyema necessitans (EN) is a rare condition characterized by the accumulation of pus in the extra-thoracic soft tissue due to decompression of intrathoracic empyema by extending through the parietal pleura and chest wall usually by *Mycobacterium tuberculosis*. This is a report of a patient with poorly controlled diabetes mellitus type 2 presenting with high-grade fever, productive cough, dyspnoea and a left-sided pleuritic chest pain complicated by a left pleural effusion. It further extended to extra-thoracic soft tissue space without fistulation to the external environment. Non-specific signs coupled with the paucibacillary status of extrapulmonary tuberculosis may obscure the diagnosis of EN in extrapulmonary tuberculosis. However, this patient had greatly an exudative pleural effusion with raised adenosine deaminase levels highly suggestive of extrapulmonary tuberculosis. The patient underwent surgical clearance and insertion of a thoracotomy tube. He was commenced on antitubercular medication for which he had a successful recovery.

## Introduction

Empyema necessitans (EN) is a rare entity of an intrathoracic collection of pus that decompresses by extending through the parietal pleura and a weakness in the chest wall resulting in the accumulation of pus in the extra-thoracic soft tissues [1]. Thus a sinus track is generated to relieve the tension. *Mycobacterium tuberculosis* and *Actinomyces israelii* commonly give rise to pleural effusion with EN [1]. Other notable organisms include *Staphylococcus aureus* and *Streptococcal *sp. Pleural fluid analysis aids in the diagnosis and selection of appropriate antibiotics. The lack of contemporary diagnosis facilities for tuberculosis poses a huge challenge in distinguishing between tuberculous and nontuberculous empyema, particularly in resource-poor nations [2]. Antibiotics, tubal drainage, and decortication which obliterates the cavity and restores pulmonary function are treatment modalities of this illness [2]. This is a case report of a patient with tuberculosis in the background of poorly controlled diabetes mellitus type 2.

## Case presentation

A 75-year-old male patient with poorly controlled type 2 diabetes mellitus, hypertension, dyslipidemia and ischaemic heart disease presented with a fever of four days duration. The fever was of high grade and associated with chills, rigors and constitutional symptoms. He also complained of productive cough and shortness of breath associated with left-sided pleuritic-type pain. He was dyspnoeic at rest with a respiratory rate of 30/min and pulse oximetry of 98% on room air. The blood pressure was 120/80 mmHg and the pulse rate was 98/min. There were no cervical or axillary lymphadenopathy. The trachea was not deviated although the chest movements were reduced in the left upper and middle zones. There were scattered coarse crepitations in the same region. 

There was neutrophil leucocytosis with normal haemoglobin levels. His inflammatory markers were elevated. The liver biochemistry and renal profile were normal (Table 1). The blood culture and sputum for pyogenic culture did not yield any growth. His chest x-ray revealed left side upper and middle zones opacities. The patient was commenced on intravenous piperacillin-tazobactam 4.5 g eight hourly empirically for a left-side lobar pneumonia with supportive management.

**Table 1:** Laboratory investigations of the patient

Parameters	Reference Range	Day 1	Day 2	Day 5	Day 9
White blood cells	4-10 x 10^9^/L	12.14	14.9	14.1	10.6
Haemoglobin	12-16 g/dL	13.0	11.9	11.5	11.7
Platelet count	150-400x10^9^/L	284	197	234	207
Aspartate transaminase (AST)	<35 U/L	23		32	
Alanine transaminase (ALT)	<35 U/L	25		31	
Total protein	6-8 g/dL			6.3	
Serum albumin	3.5-5.0 g/dL			3.0	
Serum creatinine	74-110 µ/L	97	89	60	82
Sodium	136-146 mmol/L	137	134	141	139
Potassium	3.5-5.1 mmol/L	3.9	3.6	4.1	3.9
C reactive protein (CRP)	0-5 mg/L	>310		180	74
Erythrocyte sedimentation rate (ESR)	<30	80			
HbA1c (glycosylated haemoglobin)	<6.5%	9.5%			
Serum lactate dehydrogenase (LDH)	140 to 280 U/L			202	
Human immunodeficiency virus (HIV) antigen and antibodies				Negative	

**Figure 1:**
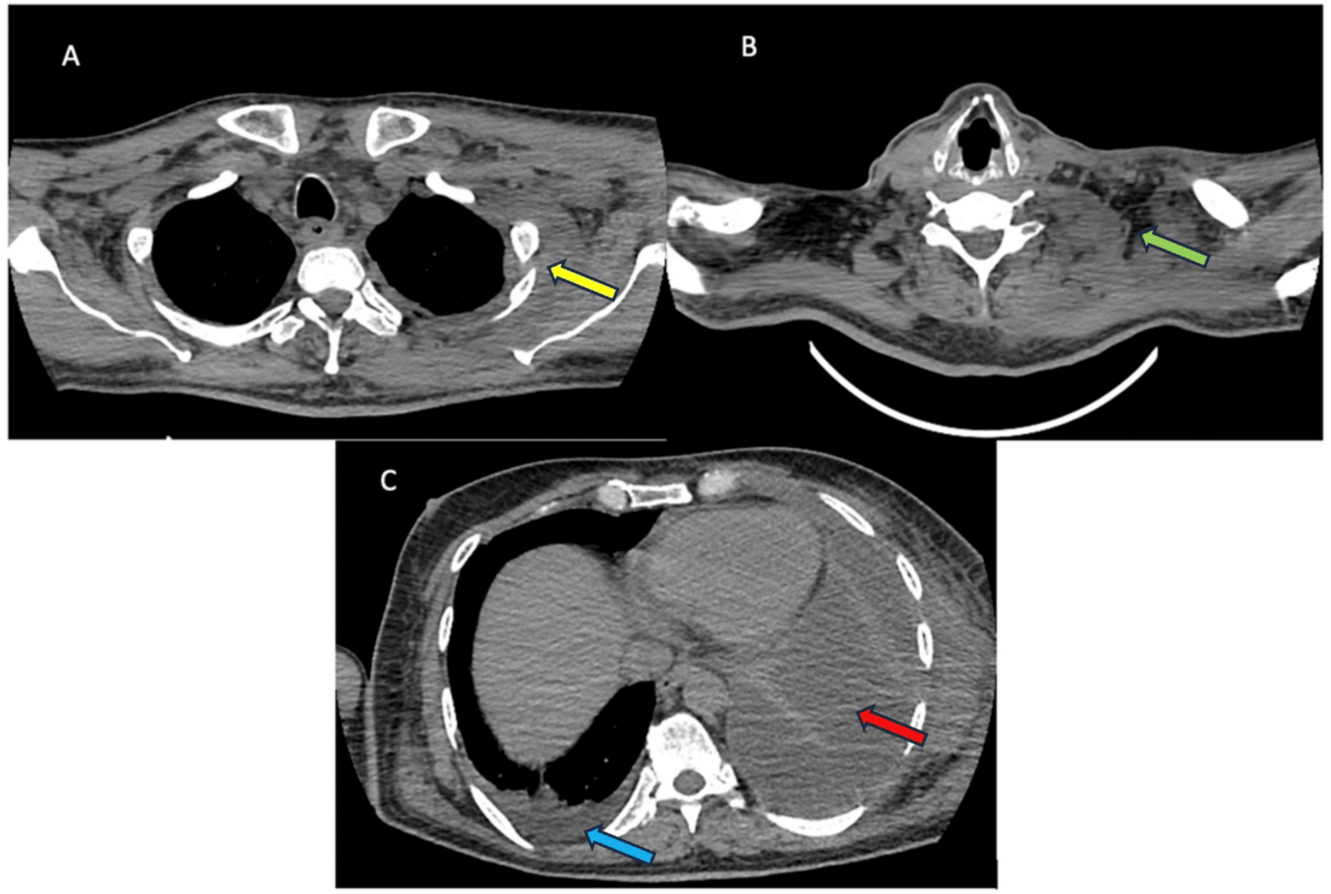
CECT chest and neck Demonstration of left-sided loculated effusion (red arrow) (c) with extension into the upper lateral chest wall (yellow arrow) (a), necrotic left cervical lymphadenopathy (green arrow) (b) with mild right-sided pleural effusion (blue arrow) (c). CECT: Contrast-enhanced computed tomography

On the fifth day of admission, he complained of left-sided neck pain and swelling. There was left supraclavicular fullness for which ultrasonography demonstrated left-side level 1-2 submandibular lymphadenopathy with surrounding inflammation. Ultrasonography of the chest revealed left-sided multiloculated effusion with collapsed consolidation. The sputum for gram stain and culture as well as acid-fast bacilli was negative. The Mantoux test was positive at 11 mm. The pleural fluid analysis was suggestive of an exudative effusion (empyema as pH was 7.2) (Table 2). Although the pleural fluid cultures for bacteria and tuberculosis were sterile and the smear for acid-fast bacilli was negative, the adenosine deaminase levels (ADA) were significantly high with the lactic acid dehydrogenase to adenosine deaminase (LDH:ADA) ratio was 3.26, suggestive of tuberculosis (the latter being <16.2). Furthermore, the GeneXpert® (Cepheid, Sunnyvale, CA) test, a cartridge-based nucleic acid amplification test (CB-NAAT) for rapid tuberculosis diagnosis was positive. Subsequently, he underwent a contrast-enhanced computerized tomography (CECT) scan of the chest, which revealed left-sided loculated effusion with extension into the upper lateral chest wall, necrotic left cervical lymphadenopathy with mild right-sided pleural effusion (Figure 1).

**Table 2:** Pleural fluid analysis done on day 5 of admission LDH: lactate acetate dehydrogenase; ADA: adenosine deaminase

Parameters	Reference Range	Result
Colour		Yellowish
pH		7.2
Macroscopy		Turbid
Protein		4.5 g/dL
Red cells		20-25/hpf
Pus cells		10-15/hpf
Cytology		No atypical or malignant cells seen
Bacteria (Gram Stain)		Negative
LDH	140-280 U/L	986.3
ADA	<40 U/L	302
Bacterial culture		Negative
Tuberculosis culture		Negative
GeneXpert® test		Positive

As there was clinical and radiological evidence of tuberculosis for EN, the patient underwent surgical drainage of pus, decortication of the pleura and application of a thoracotomy tube, which was kept in situ for 10 days. He was commenced on four-drug fixed-dose combination (4-FDC) anti-tuberculosis therapy with isoniazid, rifampicin, ethambutol and pyrazinamide (HRZE) together with pyridoxine (vitamin B6) for two months followed by isoniazid and rifampicin for four months. Nutritional and glycaemic optimization was carried out. He was reviewed at the chest clinic and had good adherence to anti-tuberculosis therapy (ATT). The surgical site healed well without recollection of purulent material in intra- and extra-thoracic spaces. The patient established full recovery with no complications during outpatient visits even one year after the first presentation. His review of haematological and biochemical investigations were normal and the repeat contrast-enhanced computed tomography (CECT) scan of the chest demonstrated a resolution of the inflammatory changes.

## Discussion

Extrapulmonary tuberculosis accounts for 15% of all tuberculosis cases [3]. Pleural effusion associated with EN is a cause of higher morbidity and mortality. Abscesses are caused by chronic pleural inflammation, which begins as empyema and progresses to a bronchopleural fistula, which causes substance leakage to the chest wall [4]. This inflammatory process can cause unspecified clinical symptoms for years in both immunocompromised and immunocompetent individuals. Tuberculous EN notably is associated with a mass formation on the chest. This patient had poorly controlled diabetes mellitus, which indicates an immunocompromised state.

It is well-recognized that symptoms of pulmonary tuberculosis may be obscured in the older adult population. The lack of clinical signs associated with persistent tuberculous empyema is also well-known [5]. When a patient develops a bronchopleural fistula or an EN, its diagnosis may occasionally be taken into account [6]. Long-standing constitutional symptoms are also directed toward possible tuberculosis infection. Non-infectious diseases such as lymphoma and primary lung neoplasms were also taken into consideration given the chronic nature of the clinical picture of our patient. 

The clinical diagnosis is a challenging process. This is contributed by the non-specific signs and the paucibacillary status of extrapulmonary tuberculosis as in this patient whose microbiological specimens were negative. However, he had strong evidence of an exudative pleural effusion as per Lights criteria (an empyema with a pH of 7.2) and a high likelihood of tuberculosis with significantly high ADA levels and positive GeneXpert® test (a cartridge-based nucleic acid amplification test). Furthermore, the LDH:ADA ratio being less than 16.2 was also in favor of the aetiology being tuberculosis [7]. CECT chest imaging is diagnostic for EN, which shows pleural effusion with an extra-pleural mass of the chest wall or soft tissues. Other CT scan abnormalities that could be seen include rib destruction, pleural thickening, soft tissue swelling, and/or calcification [8].

EN treatment consists of a combination of surgical and medical treatment. Antitubercular therapy (ATT) is usually sufficient to prevent relapses without the need for surgery. Most guidelines recommend six months to a year of ATT. Surgical treatment includes video-assisted thoracoscopic surgery (VATS), open thoracotomy and drainage, debridement and lung resection [9]. Our patient responded well to conventional antituberculous medication combined with surgical drainage with thoracic tube placement. He did not require further invasive procedures. EN can recur due to inadequate antimicrobial treatment or suboptimal drainage. Hence, it is important to treat with optimal antimicrobial therapy with appropriate surgical interventions where necessary. Patients with immunocompromised states such as HIV or malignancy and poorly controlled diabetes mellitus are at greater risk of EN.

## Conclusions

Empyema necessitans (EN) is a rare disease entity which has serious consequences if left untreated. Clinicians should have a high index of suspicion in those with poor glycaemic control and in settings where the disease burden of tuberculosis is high. Prompt diagnosis and treatment will improve the morbidity and mortality of the illness.
